# Targeting NF-κB with Nanotherapy in a Mouse Model of Adult T-Cell Leukemia/Lymphoma

**DOI:** 10.3390/nano11061582

**Published:** 2021-06-16

**Authors:** Daniel A. Rauch, John C. Harding, Lee Ratner, Samuel A. Wickline, Hua Pan

**Affiliations:** 1 Department of Medicine, Division of Molecular Oncology, Washington University School of Medicine, St. Louis, MO 63110, USA; jharding@wustl.edu (J.C.H.); lratner@wustl.edu (L.R.); 2 USF Health Heart Institute, University of South Florida, Tampa, FL 33602, USA; wickline@usf.edu

**Keywords:** adult T-cell leukemia/lymphoma, human T-cell leukemia virus type 1, NF-kB activation, p5RHH, siRNA delivery

## Abstract

Adult T-cell leukemia/lymphoma (ATLL) is an aggressive, clonal malignancy of mature T cells caused by human T-cell leukemia virus type 1. Although it is a rare tumor type, it serves as an excellent model of a virus driven process that transforms cells and engenders a highly malignant tumor that is extraordinarily difficult to treat. The viral transcriptional transactivator (Tax) in the HTLV-1 genome directly promotes tumorigenesis, and Tax-induced oncogenesis depends on its ability to constitutively activate NF-κB signaling. Accordingly, we developed and evaluated a nano-delivery system that simultaneously inhibits both canonical (p65) and noncanonical (p100) NF-κB signaling pathways locally in tumors after systemic administration. Our results demonstrate that siRNA is delivered rapidly to ATLL tumors after either i.p. or i.v. injection. The siRNA treatment significantly reduced both p65 and p100 mRNA and protein expression. Anti-NF-κB nanotherapy significantly inhibited tumor growth in two distinct tumor models in mice: a spontaneous Tax-driven tumor model, and a Tax tumor cell transplant model. Moreover, siRNA nanotherapy sensitized late-stage ATLL tumors to the conventional chemotherapeutic agent etoposide, indicating a pleiotropic benefit for localized siRNA nanotherapeutics.

## 1. Introduction

Adult T-cell leukemia/lymphoma (ATLL) is an aggressive, clonal malignancy of mature T cells caused by human T-cell leukemia virus type 1 (HTLV-1) [1]. While approximately 20 million people are infected worldwide, HTLV-1 is rare in the US (approx. 1000 cases/year) [2,3]. When the virus is acquired during infancy, ATLL manifests after a 30–60 year latency as an aggressive malignancy in 3–8% of infected individuals [4]. This disease is refractory to therapy with a mean survival of 1–3 years after diagnosis. Although the molecular mechanisms driving ATLL are known, ATLL patients are not yet receiving tailored treatment but instead are treated by approaches used for non-Hodgkin lymphoma entailing conventional chemotherapy, interferon-α, antiretroviral therapy, stem cell transplantation, antibodies, or combination approaches [5,6,7,8,9]. Without directly targeting fundamental molecular mechanisms of ATLL, the benefits of current treatment regimens remain modest.

In the HTLV-1 genome, a 40 kDa viral transcriptional transactivator (Tax) is the oncoprotein that promotes tumorigenesis. Tax-mediated oncogenesis involves abnormal constitutive activation of both canonical and non-canonical NF-κB signaling pathways [10,11,12,13,14,15]. The critical dependence of Tax-associated oncogenesis on NF-κB activation has been confirmed by examining HTLV-1 molecular clones harboring specific Tax mutations. The mutation of G137A/L138S [16] in the Tax gene, which eliminates the ability of Tax to activate NF-κB signaling, abolishes the ability of the virus to transform primary cells [14,16,17,18]. While the L319R/L320S [16] mutation, which suppresses the ability of Tax to activate transcription via the CREB pathway without altering NF-κB signaling, retains immortalization activity [16,17,18]. 

The NF-κB pathway is constitutively activated in HTLV-1 infected cells and is a central driver in ATLL. This pathway regulates well over 100 target genes, many of which participate in host immunity, and multiple steps during oncogenesis [19]. NF-κB is regulated via both Tax dependent and Tax-independent mechanisms including constitutive activation of TCR signaling, which is a hallmark of ATLL [15,20,21]. The NF-κB-IRF4 transcriptional regulatory network in mature T cells forms a coherent feed-forward loop in ATLL that promotes lymphocyte proliferation, activates super enhancers, and regulates critical oncogenes [22]. Accordingly, ATLL is one of many hematopoietic malignancies for which an NF-κB-targeted therapy could provide a significant clinical benefit. This evidence serves as a proof-of principle of NF-κB as a therapeutic target that warrants further consideration. 

The NF-κB family has five transcriptional activator members: p65 (Rel A), Rel B, c-Rel, p50 (NF-κB1), and p52 (NF-κB2), which can form different homo- or heterodimers to effect myriad cellular functions [23,24]. Under physiological conditions, NF-κB dimers are retained in the cytoplasm in an inactive complex with their inhibitor proteins (IκB), or as protein precursors (p100) [24,25,26]. In response to stimuli, NF-κB dimers are released to translocate to the nucleus after IkB degradation (canonical pathway) or p100 (non-canonical pathway) [24,26,27]. 

In ATLL cells, Tax directly induces NF-κB nuclear translocation by activating the IκB Kinase (IKK) complex [28] and processing p100 to produce p52 [29,30,31]. ON activation of the canonical NF-κB signaling pathway, IκB proteins are phosphorylated by activated IKK complex [24,32]. Consequently, liberated NF-κB heterodimers, p65/p50, undergo nuclear translocation to induce gene expression. In the activation of canonical NF-κB signaling, it has been demonstrated that Tax physically interacts with IKKγ, the regulatory subunit of the IKK complex, to maintain chronic NF-κB activation [28]. A leucine-rich region of Tax interacts with two leucine zipper domains within IKKγ. One is located between residues 100 and 140, and the other is located between residues 312 and 336 [28]. Moreover, new results reported in a recently published article indicated that the antigen-mediated triggering of B cell receptor (BCR) can activate and causing nuclear translocation of p50/p65 complex in as little as 30 s. This alternative BCR activated NF-κB signaling pathway activation is IKK independent [33]. 

For non-canonical NF-κB signaling pathway, activation depends on inducible p100 processing [31]. Moreover serving as p52 precursor, p100 also regulates cellular and transcriptional activities [34,35] of RelB, which forms heterodimers with p52 [36]. Moreover, p100 also plays an important role in RelB stabilization. Without p100, the level of RelB is significantly reduced [37]. Under normal physiological conditions, cellular p100 processing is tightly regulated and it is largely present in its unprocessed form [29]. However, in HTLV 1 infected or Tax expressing cells, high cellular levels of p52 indicate increased p100 expression and processing. It has been documented that Tax not only up-regulates p100 expression, but also enhances p100 processing [38,39]. Physical interactions between Tax and p100 are essential for Tax-induced activation of the non-canonical NF-κB signaling pathway [38,39,40,41]. Tax also enhances p100 processing by promoting recruitment of IKKα to p100 for its phosphorylation and ubiquitylation [29] and up-regulation of p100 expression [38,39]. 

In this study, we targeted both NF-κB canonical (p65) and noncanonical (p100) pathways in two distinct tumor models to define the utility of systemically delivered siRNA to exert focal control of ATLL at the tumor site. To this end, we recently reported a unique peptide-based nanoparticle design for the robust systemic delivery to cells of oligonucleotides that inhibit the translation of myriad mRNA’s including NF-κB [42,43,44,45,46,47,48,49,50,51,52,53,54]. This peptide construct is based on selective modifications to a 26-amino acid cationic, amphipathic natural peptide, melittin. This modified peptide construct essentially eliminates its cell membrane disrupting toxicity in the systemic circulation yet retains its ability to rapidly condense anionic siRNA into a 55 nm particle in a simple and rapid mixing procedure. The nanoparticle format protects the siRNA from destruction in the circulation, facilitates cellular uptake by macropinocytosis, and promotes simultaneous endosomolysis and RNA release into the cytoplasm to engage the RISC complex for targeted mRNA destruction [52,53,54]. 

We previously showed that with TAX-transformed F8 cells from a murine model of HTLV-1-induced ATLL that nanoparticle siRNA targeting both p65 and p100/52 in vitro yields additive suppressive effects on malignant cell growth [52]. In this study, we now validate the efficacy of NF-κB siRNA nanoparticles in two ATLL animal models in vivo. The first model is the spontaneous transgenic immunocompetent TAX-LUC mouse where tumorigenesis is driven by inflammation, and thus, allows interrogation of NF-kB activation in both pre-malignant lesions and tumors [55]. The second is a primary allograft model, in which primary tumor homogenates from spontaneously arising TAX-LUC tumors are engrafted into Beige/Nude/Xid (BNX) mice where tumor growth and size are NF-κB dependent. Our results demonstrate that this anti-NF-κB nanotherapy effectively inhibits ATLL tumor growth and sensitizes late-stage ATLL tumors to chemotherapy. 

## 2. Materials and Methods 

### 2.1. Preparation of p5RHH-NF-kB Inhibitory Polyplex Nanoparticles

These peptide-based self-assembling RNA polyplex structures were previously described in detail [52,53]. In brief, a custom-designed RNA condensing peptide, p5RHH (VLTTGLPALISWIRRRHRRHC, Genscript, Piscataway, NJ, USA), was prepared at 20 mM in DNase-, RNase-, and protease-free sterile water (Cellgro at Corning, Tewksbury, MA, USA) and stored at −80 °C before use. p65 and p100 siRNAs were purchased from Sigma (St. Louis, MO, USA) and dissolved in 1× siRNA buffer (prepared from 5× siRNA buffer Dharmacon, Inc, Lafayette, CO, USA) at 100 mM, aliquoted, and stored −80 °C before use. p5RHH-NF-κB inhibitory nanoparticles were formulated at peptide:siRNA ratio of 100:1 and incubated on ice for 10 min for either i.v. or i.p. injection at a dosage of total siRNA of 0.5 mg/kg. 

### 2.2. Real-Time RT-PCR

Total RNA was extracted from excised tumors with an RNeasy Mini Kit (Qiagen, Valencia, CA, USA). Primers were purchased from Qiagen (Valencia, CA, USA). Real-time RT-PCR was performed on an ABI 7300 system (Applied Biosystems, Foster City, CA, USA).

### 2.3. Western Blot

A protein extraction buffer was prepared by adding one protease inhibitors (Sigma, St. Louis, MO, USA) per 10 mL Radioimmunoprecipitation assay (RIPA) buffer (Sigma, St. Louis, MO, USA) with phenylmethylsulfonyl fluoride (Cell Signaling Technology, Danvers, MA, USA) at a final concentration of 1 mM. Two volumes of the protein extraction buffer were added to 1 volume of tumor and homogenization was performed with a Bullet Blender Storm (Next Advance, Troy, NY, USA), followed by 10 min centrifugation at 12,000× *g* at 4 °C. Protein concentration was quantified with BCA protein assay (Thermo Fisher Scientific, Tampa, FL, USA). Under reducing conditions, total protein was fractionated using sodium dodecyl sulfate polyacrylamide-gel electrophoresis. Membranes were probed with rabbit anti-p65 or anti-p100 (1:1000 dilution, Cell Signaling Technology, Danvers, MA, USA) and rabbit anti-beta actin (1:1000 dilution, Sigma, St. Louis, MO, USA). Membranes were washed and incubated with secondary antibody anti-rabbit HRP (1:10,000 dilution, Santa Cruz Biotechnology, Dallas, TX, USA). Bands were visualized with Pierce ECL Western blotting substrate (Thermo Fisher Scientific, Tampa, FL, USA) using ChemiDoc MP (Bio-Rad Laboratories, Hercules, CA, USA). Knockdown of proteins was quantified using ImageJ (National Institutes of Health, Bethesda, MD, USA).

### 2.4. Histology

Extracted tumors were fixed in 10% formalin (Thermo Fisher Scientific, Tampa, FL, USA) for over 24 h and less than 48 h before tissue processing and paraffin embedding. Serial sections were collected from tumor paraffin blocks for hematoxyln and eosin (H/E) staining. Stained slides were then imaged at both 4× and 40×. 

### 2.5. IVIS Imaging

A Xenogen IVIS Spectrum imaging system (Caliper LifeSciences, Hopkinton, MA, USA) was used for in vivo fluorescence imaging. Mice were maintained under isoflurane inhalation anesthesia during the image acquisition. Fixed settings (excitation, 500 nm; emission, 540 nm; exposure time, 0.5 s; binning factor, 8; f value, 2; and field of view, 12.9) were used for all imaging acquisitions. 

### 2.6. Animal Studies

Animal studies were performed in accordance with research protocols approved by the Division of Comparative Medicine (DCM) and the Institutional Animal Care and Use Committee (IACUC; Animal Welfare Assurance #A-3381-162 01) at Washington University in Saint Louis. TAX-LUC transgenic mice have been described [14]. For allograft experiments, peripheral tumors excised from TAX-LUC mice were homogenized aseptically, resuspended in sterile saline, and injected (5 million cells, subcutaneously) into BNX recipients. Tumor and spleen measurements were obtained with calipers. Whole blood collected in heparin tubes was submitted to DCM for automated and manual cell counts of peripheral blood lymphocytes. 

### 2.7. Statistics

Unpaired, two-tailed Student’s *t*-tests were performed to assess differences between groups unless indicated otherwise. Significance of difference was established at *p* > 0.05 or better.

## 3. Results and Discussion

### 3.1. p5RHH-Based siRNA Delivery Platform Delivers siRNA to the Tumor Site after Systemic Administration

**Figure 1:**
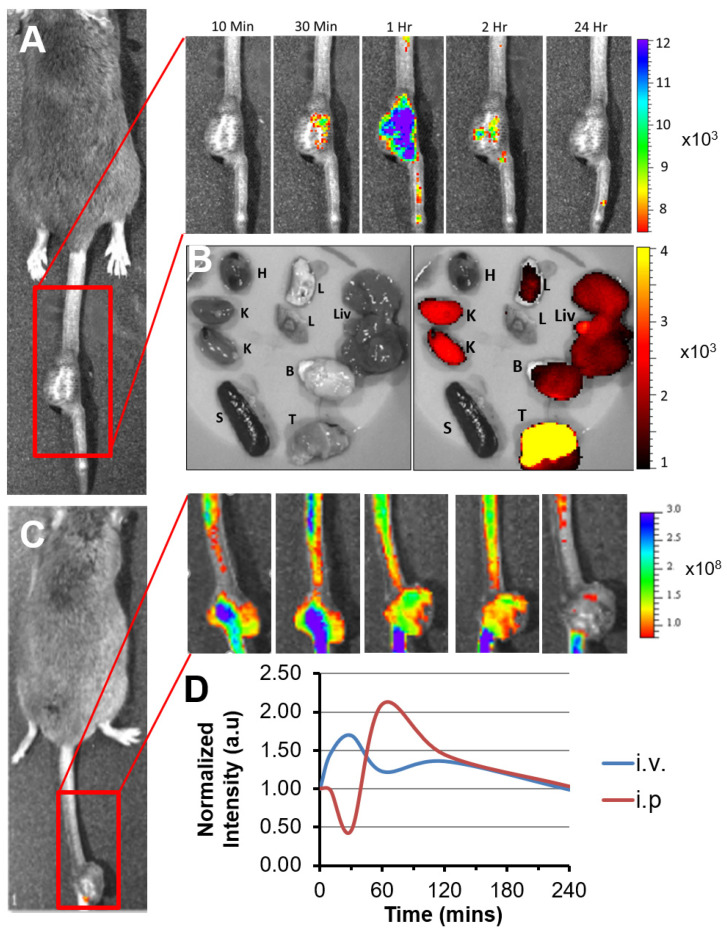
p5RHH platform delivers siRNA to the tumor after systemic administration. (**A**) Representative images of siRNA delivery by p5RHH in the tumor over time after i.p. injection. (**B**) Representative images of siRNA delivery by p5RHH to the vital organs and tumor 24 h post i.p. injection (H: Heart, K: Kidneys, S: Spleen, L: Lung, B: Brain, Liv: Liver, T: Tumor) (left), bright field image and (right), fluorescence superimposed on the bright field image. (**C**) Representative images of siRNA delivery by p5RHH in the tumor over time after i.v. injection. (**D**) Kinetics of siRNA delivery to the tumor by p5RHH via either i.v. or i.p. injection. Heat map scale units for (**A**–**C**) is (photons/sec/cm^2^/steradian)/(μW/cm^2^).

Recently, we reported a peptide(p5RHH)-based nucleic acids delivery platform, which protects nucleotides from serum degradation with circulation t_1/2_ ≈ 159 min [43], enables cytoplasmic delivery through endosomal escape via its pH sensing capability [52], and exhibits a benign safety profile without alteration of systemic innate or adaptive immunoresponsiveness, or cardiac toxicity [43,47,51]. In a previous in vitro study [52], we demonstrated that the p5RHH-based delivery platform is capable of specific inhibition of both canonical and noncanonical NF-κB signaling pathways in transformed F8 lymphoma cells [56] using simultaneously delivery of both p65 and p100 siRNAs. To determine if p5RHH-based nucleic acids delivery platform is capable of delivering siRNA to tumors in vivo after systemic administration, TAX-LUC mice (*n* = 4) received intraperitoneal or intravenous administration of p5RHH-based nanoparticles loaded with Cy5.5-conjugated siRNA. A time course of fluorescent images of TAX-LUC mice demonstrated rapid uptake of siRNA in tumors (Figure 1). Delivery kinetics after intraperitoneal administration peaked at 60 min post injection (Figure 1A,D), whereas intravenous administration of p5RHH-Cy5.5siRNA nanoparticles resulted in more rapid distribution of fluorescence, peaking around 30 min post injection (Figure 1C,D). Both routes of administration consistently delivered siRNA to tumors in vivo, likely due to leaky vasculature in the tumor through the endothelial permeability and retention (“EPR”) effect (Figure 1B). Moreover, the prominent siRNA signal detected in tumor, the high signals in kidney are consistent with our previous publications that major clearance route of this nanoparticle is kidney [51]. We also detected some siRNA signal in the liver, lung, and brain, which may be due to lymphoma associated inflammation in central nervous system [57,58,59,60,61] and other organs. Because our previous studies have shown that this delivery system spares health brain [45], lung [50], and other organs/tissues, and is not cleared appreciably by a healthy liver [51], the present results support the observation that this nanomaterial permeates leaky vasculature associated with tumoral and inflammatory microenvironment. 

### 3.2. Spontaneous Tumor Growth Is Suppressed by NF-κB Inhibitory Therapy during and after Treatment

**Figure 2:**
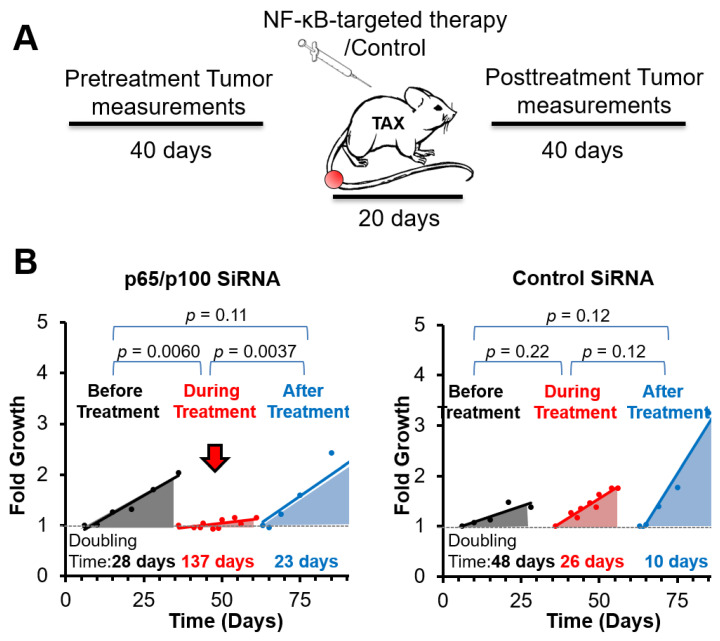
Spontaneous tumor growth is suppressed by NF-kB targeted therapy. (**A**) Scheme of tumor growth monitoring. (**B**)Tumor growth doubling time before, during, and after treatment of NF-κB inhibitory siRNA nanoparticle treated group (**left**) or control siRNA nanoparticle group (**right**).

To evaluate the impact of NF-κB inhibitory siRNA nanoparticles or control siRNA nanoparticles on tumor development in the spontaneous model, we tracked tumor size 40 days before the initiation of the treatments and then monitored tumor growth for another 40 days (Figure 2A). To determine if p5RHH nanoparticles loaded with siRNAs against p65 and p100 were capable of suppressing tumor growth in vivo, TAX-LUC transgenic mice received nanoparticles (3 i.p. injections per week for 3 weeks) dually loaded with p65/p100 siRNAs (*n* = 5) or saline as control (*n* = 7). Tumor measurements were obtained before, during, and after treatment. Growth rates and doubling times were calculated (Figure 2B). Tumor growth in mice that received p65/p100 siRNA was significantly reduced during treatment compared to control mice and compared to growth rates before and after treatment. To enhance stringency, we monitored tumor doubling times and assigned the faster growing tumors with calculated doubling times of 28 days to the treatment group. In the control group tumor doubling time was 48 days before the treatment. During the period of NF-κB inhibitory therapy, the tumor doubling time in the treatment group extended to 137 days. These results suggested that NF-κB inhibitory therapy reduced tumor growth by 80%. During the same period, the tumor doubling time in the control group was 26 days, indicating faster tumor growth in the absence of NF-κB inhibitory treatment.

These results are consistent with the mechanistic hypothesis that NF-κB signaling pathway activation is a critical determinant of ATLL tumorigenesis and growth. Accordingly, when NF-κB pathways were knocked down, the growth of the ATLL is inhibited [62]. Intriguingly, after stopping NF-κB inhibitory therapy, tumor growth recommenced to a doubling time of 23 days for the treatment group, whereas the control group accelerated even further to a doubling time of 10 days. These results illustrate that NF-κB inhibitory therapy successfully suppresses ATLL growth while also changing the natural history of subsequent tumor progression even off therapy.

### 3.3. NF-κB Inhibitory siRNA Nanoparticles Suppress Growth of Tumor Cell Transplants and Blood and Spleen Involvement in BNX Mice 

**Figure 3:**
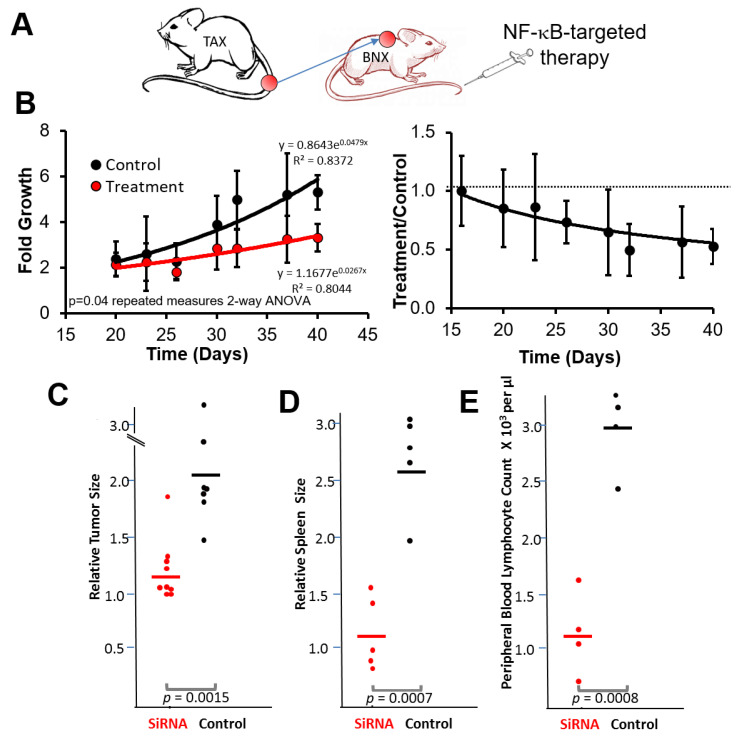
Anti-NF-kB siRNA nanoparticles suppress growth of transplanted tumor cells and lymphocyte expansion in blood and spleen. (**A**) BNX mice injected subcutaneously with tumor cells from primary TAX-LUC tumors were treated for 5 weeks (2 intravenous injections per week) with p65/p100 siRNA loaded nanoparticles or free siRNAs. (**B**) Tumor growth was measured over the course of 40 days and at necropsy. In the left panel, tumor growth rate (fold growth) is plotted over the treatment period (X-axis in days). In the right panel, the same data plotted as a ratio of treatment / control group indicates a sustained and increasing delta in tumor progression over time. The error bars represent S. D. at each time point. For treated vs. control, 95% confidence is achieved on days 26, 32, 37, and 40. In Figures (**C**–**E**), the fold change of the tumor size (**C**) and normalized size of spleens to that of average spleen size in treated group (**D**), and the absolute peripheral blood lymphocyte count (**E**) were evaluated two days after administration of the final treatment.

BNX mice implanted with tumor cell homogenates from primary TAX-LUC tumors were treated (2 i.v. injections per week for 5 weeks) with p65/p100 siRNA loaded nanoparticles (*n* = 6) vs. free siRNAs as control (*n* = 4) (Figure 3A). Tumor size caliper measurements were obtained over the course of 40 days and at the endpoint by ultrasound. The growth rate of tumors treated with p65/p100 siRNA was significantly reduced compared to control untreated mice (Figure 3B). Moreover, tumor size, spleen size, and peripheral blood lymphocyte counts were significantly lower in treated mice compared to controls (Figure 3C–E). The fold change in tumor growth in mice treated with the NF-κB inhibitory siRNA nanoparticles was 1.35 ± 0.08, while that of control mice was 2.07 ± 0.08, *p* = 0.0015. Tumor bearing mice develop enlarged spleens. By normalizing the spleen size to the average spleen size of the treatment group (1.00 ± 0.15), the spleens in the control mice (2.29 ± 0.19) were significantly larger than that in treatment group, *p* < 0.01. Peripheral blood lymphocyte counts also were affected by NF-κB inhibitory siRNA nanoparticles such that the treatment group counts were 1.04 ± 0.30 (10^3^ cells/µL) as compared to the control group (2.84 ± 0.22 (10^3^ cells/µL), *p* < 0.01). Taken together these data demonstrated that p5RHH mediated delivery of siRNA targeting p65/p100 suppressed local tumor growth and systemic manifestations of tumor growth in vivo.

### 3.4. mRNA and Protein Knokdown of p65 and p100 in BNX Mice

**Figure 4:**
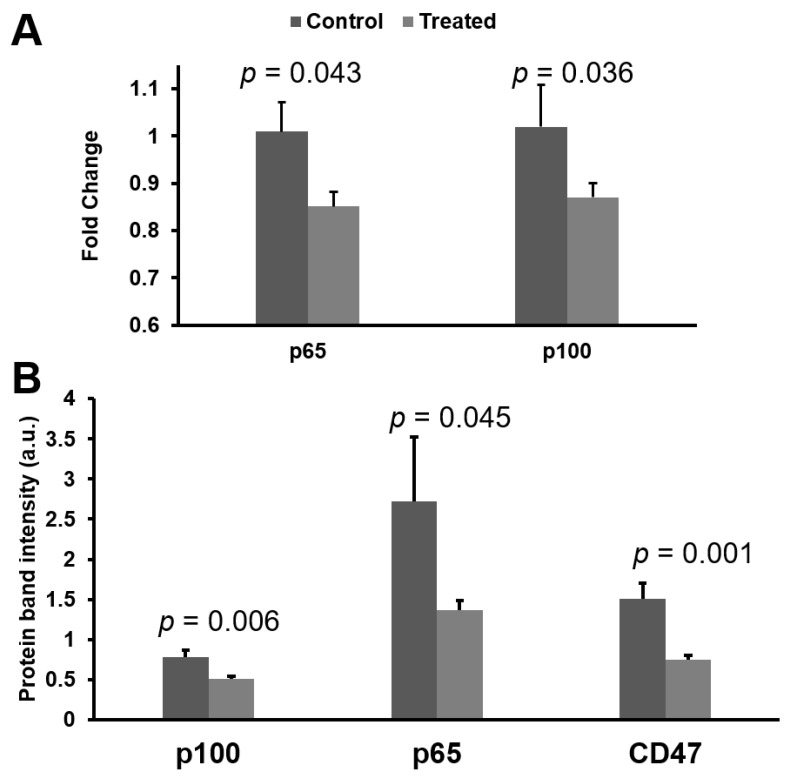
Anti-NF-κB siRNA nanoparticle treatment reduces mRNA and protein signals in vivo. (**A**) NF-kB inhibitory nanoparticle significantly reduced both p65 and p100 mRNA expression in the tumor. (**B**) NF-kB inhibitory nanoparticle significantly reduced both p65 and p100 protein expression as well as the “Do not eat me” signal CD47.

Forty eight hours after the last NF-κB inhibitory nanoparticle dose as described above, tumors were excised for evaluation of mRNA and protein expression. The RT^2^-PCR results demonstrated that NF-κB inhibitory nanoparticle loaded with both p65 and p100 siRNA significantly inhibited p65 (0.85 ± 0.03) and p100 (0.86 ± 0.03) mRNA in treated group, as compared with control group (p65: 1.01 ± 0.06, *p* = 0.043; p100: 1.02 ± 0.09, *p* = 0.032) (Figure 4A). Western blot results confirmed that nanoparticles loaded with both p65 and p100 siRNA significantly reduced p65 (p65: 1.36 ± 0.12) and p100 (0.52 ± 0.03) protein expression in the treated group, as compared with the control group (p65: 2.72 ± 0.80, *p* = 0.045; p100: 0.78 ± 0.09, *p* = 0.006) (Figure 4B). Moreover, for the nanoparticle treated mice, the CD47 (“Do not eat me”) signal was reduced (0.75 ± 0.06), as compared with that in control mice (1.51% ± 0.19) (*p* = 0.001) (Figure 4B). CD47 is an overexpressed surface protein expressed by many different types of cancer cells, including hematological malignancies [63], which prevents clearance by phagocytic cells. It has been reported that overexpression of CD47 is mediated by NF-κB in breast cancer cells [64] and glioblastoma cells [65]. Additionally, NF-κB up-regulated CD47 expression is associated with sorafenib resistance in hepatocellular carcinoma [66]. This is the first report to our knowledge that local inhibition of NF-κB in ATLL might reduce CD47 expression and could serve to prime the immune system to facilitate adjunctive anti-cancer treatments.

Although protein expression of p65 and p100 are reduced by ~50% by 48 h after the last dose, mRNA levels for p65 and p100 clearly have begun to rebound (Figure 4A). Nevertheless, the dosing scheme used in this study (2×/week) was sufficient to suppress NF-κB protein levels throughout the treatment course in order to reduce tumor growth (Figure 4B). However, the rapid up-regulation of NF-κB mRNA in this aggressive tumor model might be expected, which suggests that frequent continued dosing of NF-κB siRNA when used as a single agent might be required to maintain tumor growth suppression. Alternatively, we hypothesize below that the use of siRNA nanoparticles in concert with other combination therapies could enhance therapeutic efficacy. 

### 3.5. NF-κB Inhibitory siRNA Nanoparticles Sensitize ATLL Tumors to Adjunctive Chemotherapy 

**Figure 5:**
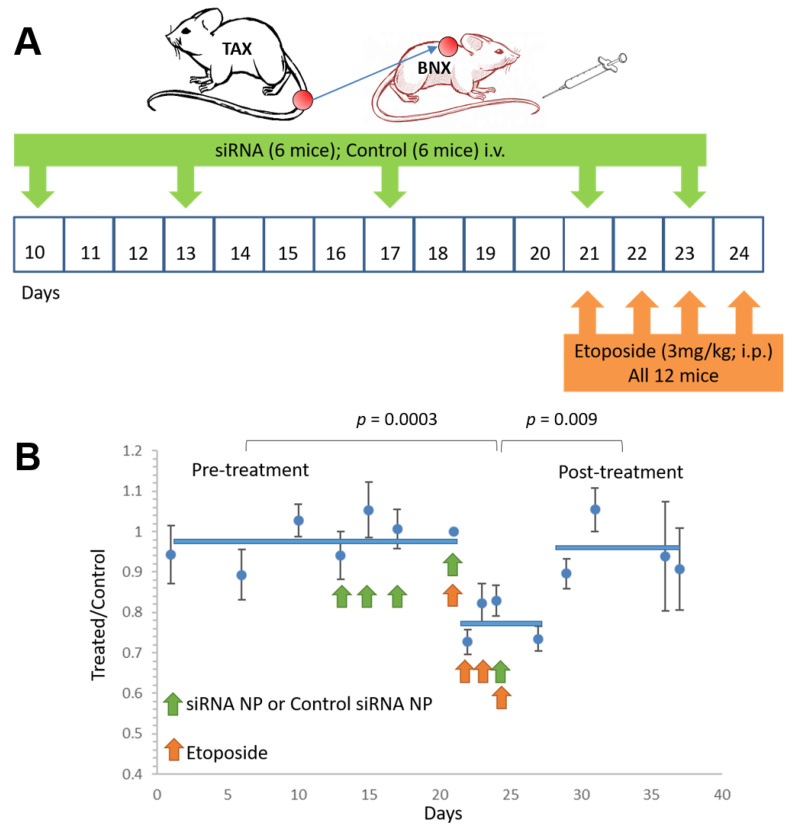
Anti-NF-kB siRNA nanoparticles sensitize tumors to traditional chemotherapy. (**A**) Scheme of treatment schedules. There are two groups with six mice each receiving NF-κB inhibitory nanoparticle with siRNA of p65 and p100, or saline injection as control and then all 12 mice were received etoposide treatment. (**B**) Tumor growth was measured in BNX mice injected subcutaneously with tumor cells from primary TAX-LUC tumors treated with saline (control) or NF-κB inhibitory nanoparticle treatment (siRNA) and then with etoposide.

To address the role of combination therapies employing siRNA inhibition, we propose the addition of etoposide, which is a standard agent for treatment of lymphoma. We sought to determine whether local delivery of anti-p65/p100 siRNAs sensitized late-stage tumors >1 cm to chemotherapy with etoposide. Tumor sizes in BNX mice bearing tumors from primary TAX-LUC mice were recorded 9 days before the mice received either NF-κB inhibitory siRNA nanoparticle or control siRNA nanoparticles by i.v. injection on days 10, 13, 17, 21, and 23 (*n* = 6 each group). All mice were then treated with etoposide (i.p.: 3mg/kg daily, on days 21–24) (Figure 5A). Tumor growth was monitored for 38 days (i.e., days 1–38). The results suggest that NF-κB inhibition renders large late-stage tumors more susceptible to chemotherapy (Figure 5B). As shown in Supplementary Figure S1, the side-by-side comparison between etoposide and etoposide with anti-NF-κB nanotherapy suggested that combined treatment reduce the tumor size, while Etoposide alone just prevented tumor growth. Since the ATLL is such an aggressive cancer, shortly after the mono- or combination therapy stopped, tumors resumed growth was observed. Our results further confirmed that ATLL is a malignancy that is highly chemotherapy-resistant with less than one-year median survival. Moreover, our recently clinical trial (NCT02631746) [67] suggested that immune checkpoint inhibitor, nivolumab, treatment caused rapid ATLL progression, despite the successes of immune checkpoint inhibitor treatment on other cancers. Therefore, it remains crucial to develop new therapeutic approaches for slowing down or reducing tumor burden. Our recently study on NF-κB downstream interferon regulatory factor 4 (IRF4) suggested that IRF4 could be a potential therapeutic target for ATLL treatment [68]. Therefore, for the further therapeutic development, it is worth considering combination therapy that targets both NF-κB and its dependent genes, such as IRF4. 

## 4. Conclusions

RNA interference induced by small interfering RNA (siRNA) has been proposed as a highly effective therapy for many diseases [69,70]. However, despite over 25 years of intense research since groundbreaking work by Tuschel et al. revealed the potential for siRNA in mammalian cells, siRNA therapeutics have demonstrated limited success in clinical applications beyond liver-related disease entities [71,72,73,74,75,76]. Systemic delivery of efficacious quantities of siRNA into the cytoplasm of targeted cells is still the major barrier to in vivo RNAi. The large molecular weight (~14kDa) and high surface charge prevent naked siRNA from passing through the cell membrane to reach cytoplasm where siRNA engages the RISC complex. These traits, combined with a serum half-life of only ~10 min for naked siRNA, necessitates the packaging of siRNA with transport and transfection agents [77]. Unfortunately, endocytic pathways present another barrier as siRNA must escape the endosomal/lysosomal compartment where it may be degraded in an acidic environment [77,78,79,80,81]. Despite these challenges, cationic lipids and polymers have experienced some success for siRNA transfection in experimental situations [70,77,78,82,83,84]. Unfortunately, these types of transfection agents can exhibit unacceptable cytotoxicity [85,86,87], due in part to aggregation with serum proteins and complement activation [88,89,90]. 

Cell-penetrating peptide (CCP)-based siRNA delivery agents have shown promise with respect to reduced cytotoxicity [91,92,93,94]. However, CCP-based siRNA delivery also suffers from challenges of endosomal trapping [91,92,95,96], which is sometimes ameliorated by chemical conjugation of CCPs to membrane active lipids or endosomolytic agents to achieve endosomal escape [97,98,99]. To overcome the aforementioned challenges of systemic nucleic acid delivery, we have developed a peptide (“p5RHH”)-based nucleotide delivery platform, which protects nucleotides from serum degradation in vivo. The circulating t_1/2_ ≈ 159 of minutes [43] permits significant accumulation of siRNA in tumors and uptake in cells by macropinocytosis, followed by pH-sensitive endosomal escape [52]. In prior reports, we have confirmed a benign safety profile without alteration of systemic innate or adaptive immunoresponsiveness, or organ toxicity [43,47,51]. 

In this study, we demonstrated that this p5RHH nucleotide delivery platform enables broad spectrum anti-NF-κB siRNA nanotherapy localizing to ATLL tumor sites that: suppress primary tumor growth, alter the natural history of subsequent tumor progression, and sensitize late-stage ATLL tumors to conventional chemotherapy. Because ATLL is highly refractory to currently available therapies and exhibits a mean survival of 1–3 years after diagnosis, the proposed siRNA nanotherapy approach might significantly improve clinical management of ATLL.

## Data Availability

The data presented in this study are available in the article or Supplementary Materials.

## Supplementary Materials

The following are available online at https://www.mdpi.com/article/10.3390/nano11061582/s1, Figure S1: Combination therapy of Etoposide and anti-NF-κB nanotherapy reduced tumor size, while monotherapy of Etoposide stopped tumor growth during the treatment period.
