# Supplementary material for: Targeting NF-κB with Nanotherapy in a Mouse Model of Adult T-Cell Leukemia/Lymphoma
Source: Nanomaterials (Basel). 2021 Jun 16;11(6):1582. doi: 10.3390/nano11061582 (PMC8234599; doi:10.3390/nano11061582)
Supplement: Supplementary file 1 [file nanomaterials-11-01582-s001.zip › nanomaterials-1245977-supplementary.pdf]

Supplementary materials

Targeting NF-κB with nanotherapy in a mouse model of Adult T-Cell Leukemia/Lymphoma

Daniel A. Rauch [1,*], John C. Harding [1], Lee Ratner [1], Samuel A. Wickline [2] and Hua Pan [2,*]

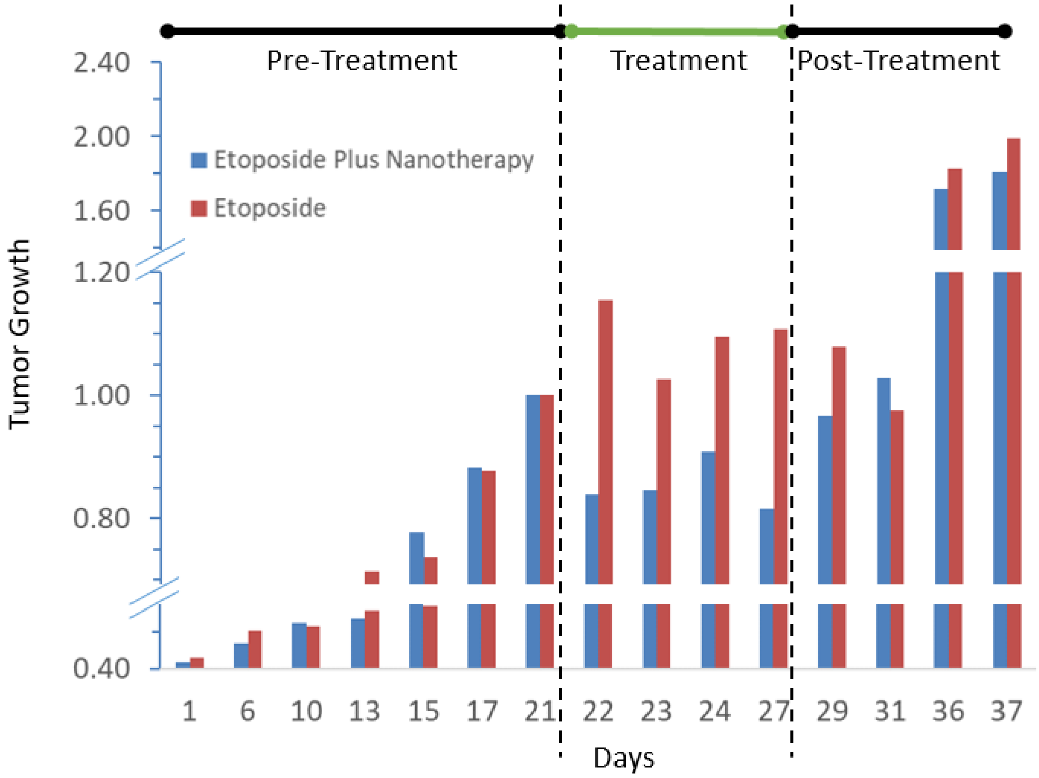

Supplementary Figure S1. Combination therapy of Etoposide and anti-NF-κB nanotherapy reduced tumor size, while monotherapy of Etoposide stopped tumor growth during the treatment period. All the tumor sizes have been nor-malized to the tumor size on day 21, when the combination therapy started.
